# Asparagine Deprivation Causes a Reversible Inhibition of Human Cytomegalovirus Acute Virus Replication

**DOI:** 10.1128/mBio.01651-19

**Published:** 2019-10-08

**Authors:** Chen-Hsuin Lee, Samantha Griffiths, Paul Digard, Nhan Pham, Manfred Auer, Juergen Haas, Finn Grey

**Affiliations:** a Division of Infection and Immunity, The Roslin Institute, University of Edinburgh, Easter Bush, Midlothian, United Kingdom; b Division of Infection and Pathway Medicine, University of Edinburgh, Edinburgh, United Kingdom; c MRC Centre for Regenerative Medicine, Institute for Stem Cell Research, School of Biological Sciences, University of Edinburgh, Edinburgh, United Kingdom; Columbia University Medical College

**Keywords:** asparagine, human cytomegalovirus, latency, antiviral, high throughput, siRNA screen, systems biology, virus

## Abstract

As obligate intracellular pathogens, viruses rely on the host cell machinery to replicate efficiently, with the host metabolism extensively manipulated for this purpose. High-throughput small interfering RNA (siRNA) screens provide a systematic approach for the identification of novel host-virus interactions. Here, we report a large-scale screen for host factors important for human cytomegalovirus (HCMV), consisting of 6,881 siRNAs. We identified 47 proviral factors and 68 antiviral factors involved in a wide range of cellular processes, including the mediator complex, proteasome function, and mRNA splicing. Focused characterization of one of the hits, asparagine synthetase (ASNS), demonstrated a strict requirement for asparagine for HCMV replication which leads to an early block in virus replication before the onset of DNA amplification. This effect is specific to HCMV, as knockdown of ASNS had little effect on herpes simplex virus 1 or influenza A virus replication, suggesting that the restriction is not simply due to a failure in protein production. Remarkably, virus replication could be completely rescued 7 days postinfection with the addition of exogenous asparagine, indicating that while virus replication is restricted at an early stage, it maintains the capacity for full replication days after initial infection. This study represents the most comprehensive siRNA screen for the identification of host factors involved in HCMV replication and identifies the nonessential amino acid asparagine as a critical factor in regulating HCMV virus replication. These results have implications for control of viral latency and the clinical treatment of HCMV in patients.

## Abstract (precis)

HCMV accounts for more than 60% of complications associated with solid organ transplant patients. Prophylactic or preventative treatment with antivirals, such as ganciclovir, reduces the occurrence of early onset HCMV disease. However, late onset disease remains a significant problem, and prolonged treatment, especially in patients with suppressed immune systems, greatly increases the risk of antiviral resistance. Very few antivirals have been developed for use against HCMV since the licensing of ganciclovir, and of these, the same viral genes are often targeted, reducing the usefulness of these drugs against resistant strains. An alternative approach is to target host genes essential for virus replication. Here we demonstrate that HCMV replication is highly dependent on levels of the amino acid asparagine and that knockdown of a critical enzyme involved in asparagine synthesis results in severe attenuation of virus replication. These results suggest that reducing asparagine levels through dietary restriction or chemotherapeutic treatment could limit HCMV replication in patients.

## INTRODUCTION

Human cytomegalovirus (HCMV) is a highly prevalent herpesvirus, infecting greater than 30% of the worldwide population. Although normally asymptomatic in healthy individuals, HCMV infection is a significant cause of morbidity and mortality in immunocompromised populations, individuals with heart disease, and recipients of solid organ and bone marrow transplants (1). HCMV is also the leading cause of infectious congenital birth defects resulting from spread of the virus to neonates (2).

Cellular metabolism is a tightly regulated process in mammalian cells and is often manipulated during viral infection. As obligate intracellular pathogens, viruses rely on host metabolites and often alter host metabolism to increase pools of free nucleotides and amino acids, as well as inducing fatty acid biosynthesis to aid efficient virus replication (3, 4).

Infection with HCMV has been demonstrated to alter the host cell metabolic pathways, increasing glycolysis and glutamine metabolism while maintaining protein translation through activation of mammalian target of rapamycin complex 1 (mTORC1) (5). Glucose metabolism is a key pathway to supply carbon precursors for cellular biosynthesis and energy production. Under normal conditions, glucose is used for energy generation and cellular biosynthesis, while only a small amount of glutamine is metabolized from exogenous sources. In contrast, in HCMV-infected cells, glucose is diverted away from the tricarboxylic acid (TCA) cycle into the production of lactic acid and fatty acid, while exogenous glutamine is used as the main carbon and nitrogen source. Glutamine can donate its amino group at the gamma position for *de novo* biosynthesis of nucleotides and nonessential amino acids while being converted to glutamate (6). Glutamate can be further metabolized into α-ketoglutarate via glutamate dehydrogenase, thereby providing a key intermediate for the TCA cycle, a process known as anaplerosis, which also occurs in rapidly dividing cancer cells (7).

A recent study showed that infection with HCMV results in increased metabolism of arginine, leucine/isoleucine, serine, and valine and increased secretion of alanine, ornithine, and proline, demonstrating extensive alteration of cellular amino acid metabolism during infection (8). Furthermore, HCMV manipulates cellular signaling pathways to maintain protein synthesis during amino acid starvation. Mammalian cells have two main pathways that monitor and modulate the level of intracellular amino acids: the mTOR pathway and the amino acid response (AAR) pathway. The mTOR pathway serves to ensure a sufficient level of amino acids to support protein synthesis and cell growth. Previous studies have shown that glutamine and leucine activate the mTOR pathway via glutaminolysis and mediate cellular responses to amino acids (9). Activation of mTOR ultimately leads to the phosphorylation and activation of the ribosome-associated S6 kinase, which enables higher levels of protein synthesis, while loss of mTOR signaling results in suppression of protein synthesis. However, HCMV infection can maintain mTOR activation during amino acid deprivation through viral UL38 protein binding and antagonizing the tuberous sclerosis subunit complex 2 (TSC2), a major suppressor of mTOR (10, 11). UL38 interaction with TSC2 has also been shown to have broader effects on cellular metabolism in an mTOR-independent fashion (8, 12). These findings show that regulation of amino acid metabolism plays an important role during HCMV replication.

Here, we show that asparagine synthetase (ASNS) is a critical host factor for HCMV replication following a comprehensive small interfering RNA (siRNA) screen. Knockdown of ASNS resulted in an early restriction in virus replication. However, knockdown of ASNS had little effect on herpes simplex virus 1 (HSV-1) or influenza A virus (IAV) replication, indicating that the effects of asparagine depletion were specific to HCMV and not simply due to a loss of production of asparagine-containing proteins. Furthermore, mTOR activation was maintained in infected cells following ASNS knockdown, indicating that this was not the cause of attenuated virus replication. Remarkably, the block in viral replication could be completely rescued 7 days postinfection by the addition of exogenous asparagine to the cell media. These results suggest a novel check point in virus replication regulated by intracellular asparagine levels.

## RESULTS

### High-throughput siRNA screen identified novel host factors involved in HCMV replication.

**FIG 1:**
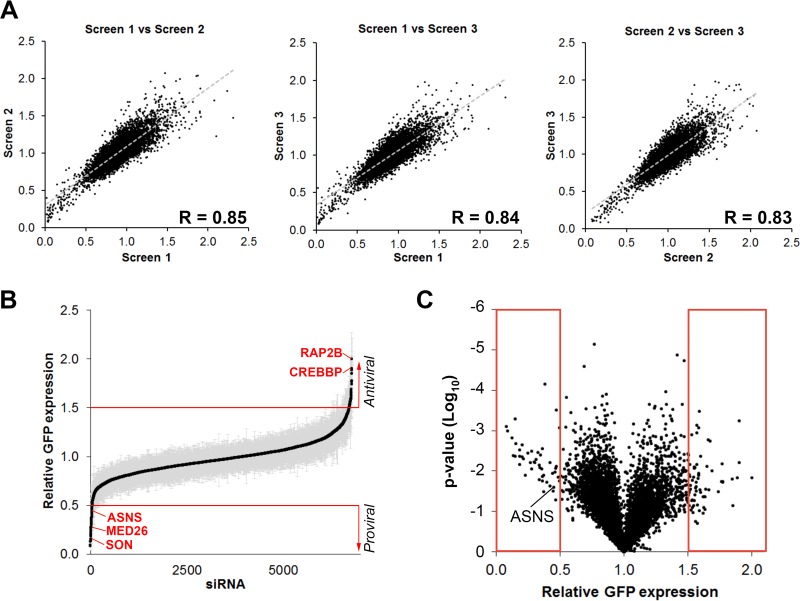
High-throughput siRNA screen identified novel host factors for HCMV replication. (A) NHDF cells were transfected with siRNA pools targeting 6,881 genes in a 384-well format. Two days after transfection, the cells were infected with HCMV TB40/E-GFP at an MOI of 5 with GFP levels monitored by plate cytometry for 7 days. Comparative analysis between three biological repeats showed high levels of correlation with Pearson coefficient scores between 0.83 and 0.85. (B) Relative GFP expression representing the level of primary replication is shown, sorted from low to high compared to control nontargeting siRNA-transfected cells. Standard deviations are shown by gray shaded area. (C) Volcano plot showing relative GFP expression versus associated *P* value. Each dot represents knockdown of a single gene. *P* values were calculated by Student’s *t* tests. Red boxes represent the top hits based on a twofold increase or decrease in relative GFP expression (listed in Table S2 and S3 in the supplemental material).

**FIG 2:**
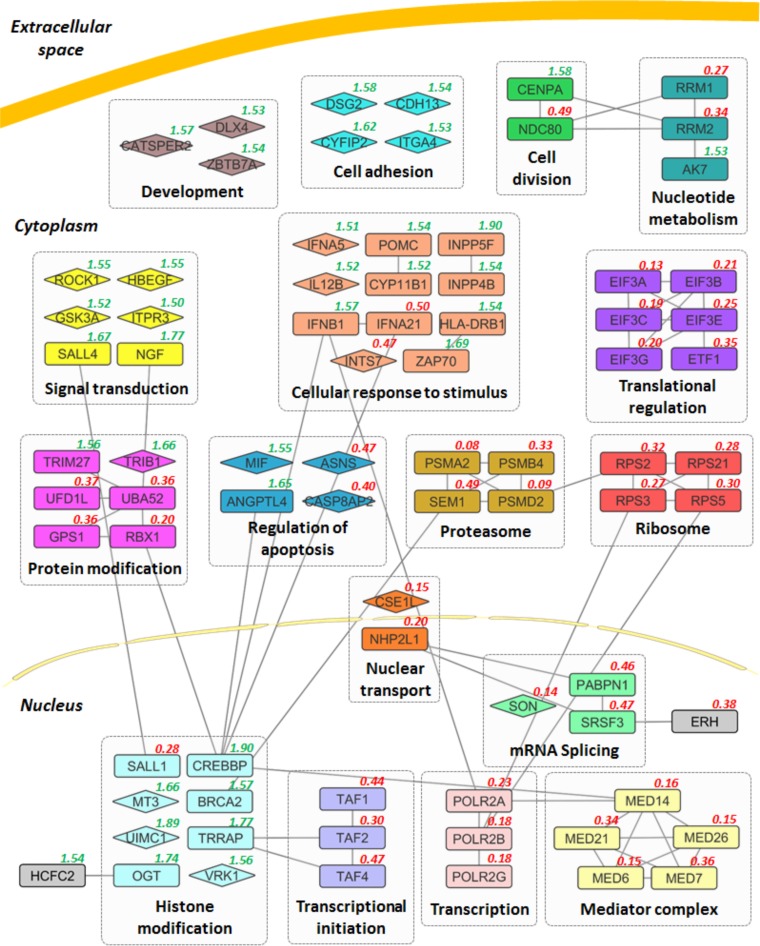
Functional analyses of top proviral and antiviral candidates. Selected candidates of the top 115 host factors are shown in functional clusters based on STRING analysis. Interactions between genes all have high interaction scores (combined score of >0.8, STRING). Genes that do not show an interaction with other genes in this figure are depicted as diamonds. Functional gene clusters are shown in their approximate cellular location with the primary replication ratio compared to negative control indicated (green indicates antiviral role, and red indicates proviral role).

To identify host factors that influence HCMV replication, a combined siRNA library comprising small interfering RNAs (siRNAs) targeting the human druggable genome, protein kinases/phosphatases, and cell cycle genes, were used in a high-throughput screen. SMARTpool siRNAs (a pool of four siRNAs per gene) targeting a total of 6,881 genes were transfected into primary normal human dermal fibroblast (NHDF) cells in a 384-well format. Cells were infected at 48 h posttransfection at a high multiplicity of infection (MOI of 5) with the low-passage-number HCMV strain TB40/E-GFP, which expresses green fluorescent protein (GFP) from a simian virus 40 (SV40) promoter (13). GFP fluorescence levels were monitored every 24 h for 7 days, with levels compared to control nontargeting siRNA-transfected cells in order to determine the effect of individual gene depletion on HCMV replication. We have previously established GFP expression as an accurate measure of early virus replication events, including viral entry, translocation to the nucleus, and viral DNA amplification, hereon referred to as primary replication. Gene expression kinetics from the SV40 promoter resembles immediate early (IE) expression kinetics, and inhibition of DNA amplification results in a loss in exponential GFP signal, likely due to decreased viral template DNA (14, 15). The assay was performed in biological triplicate with an additional replicate used to determine cytotoxicity at 7 days postinfection (DPI) (see Table S1 in the supplemental material). siRNAs were defined as cytotoxic when gene depletion led to greater than 40% cell death (106 in total; data not shown). These genes were excluded from further analyses. High correlation between biological triplicates demonstrated reproducibility within the screen (Fig. 1A). The screen identified a total of 115 host factors where knockdown led to 50% inhibition (47 proviral factors) or 50% increase (68 antiviral factors) in primary replication (Fig. 1B and C and Tables S2 and S3). Figure 2 shows a schematic summary of the top hits affecting HCMV virus replication grouped into functionally related gene clusters based on STRING analysis (16). These include members of the proteosome complex and ubiquitin-modifying factors, which have previously been shown to be important for efficient HCMV replication and the mediator complex that has been linked to immediate early transactivator function in alpha- and gammaherpesviruses (17–21). mRNA splicing factors and transcription and translation initiation factors are also identified as proviral. Interestingly, a number of histone modification factors are identified as antiviral, along with multiple signal transduction factors, developmental factors, cell adhesion factors, and factors involved in the cellular response to signaling factors. Based on the magnitude of phenotype, lack of toxicity, and novelty, nine host factors with proviral phenotypes were selected for further validation (Table 1).

**TABLE 1:**
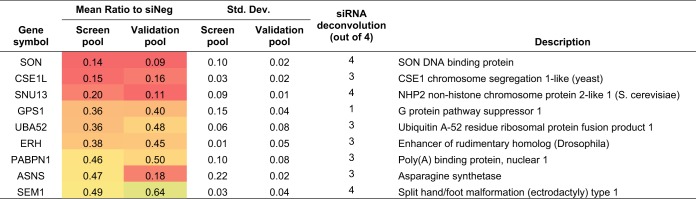
siRNA deconvolution validation of nine proviral candidates^*a*^

a Nine proviral candidates were selected for validation with deconvoluted siRNAs. Four individual siRNAs targeting different regions of each gene were used to transfect NHDF cells, followed by infection 48 h posttransfection with HCMV TB40/E-GFP at an MOI of 5. The number of deconvoluted siRNAs that showed the same phenotype as the pool is shown. An individual siRNA was considered validated when knockdown led to significant inhibition of virus replication. Colour gradient associated with mean ratio indicates level of inhibition, with highest to lowest inhibition graded from red to green. *n *=* *3.

10.1128/mBio.01651-19.4 TABLE S1 Complete data set for siRNA screen. For each gene, the mean ratio between the targeted gene and the control wells transfected with a scrambled negative siRNA are shown, along with standard deviation, cytotoxicity score, *P* value, and Z-score. *n* = 3. Download Table S1, XLSX file, 0.8 MB. Copyright © 2019 Lee et al. 2019 Lee et al. This content is distributed under the terms of the Creative Commons Attribution 4.0 International license.

10.1128/mBio.01651-19.5 TABLE S2 Proviral host factors. Host factors where knockdown results in greater than 50% decrease in virus replication based on GFP reporter expression are shown. Download Table S2, XLSX file, 0.02 MB. Copyright © 2019 Lee et al. 2019 Lee et al. This content is distributed under the terms of the Creative Commons Attribution 4.0 International license.

10.1128/mBio.01651-19.6 TABLE S3 Antiviral host factors. Host factors where knockdown results in greater than 50% increase in virus replication based on GFP reporter expression are shown. Download Table S3, XLSX file, 0.02 MB. Copyright © 2019 Lee et al. 2019 Lee et al. This content is distributed under the terms of the Creative Commons Attribution 4.0 International license.

To determine whether the observed effects on virus replication were specific to the knockdown of the identified gene and not due to artifactual off-target effects, the pools of siRNAs were deconvoluted, and each of the individual siRNAs was tested for their effect on virus replication, along with the original pool of siRNAs (Table 1). All pooled siRNAs recapitulated the phenotype in the primary screen, once again confirming the reproducibility of the assay. In eight out of nine cases, at least three of four siRNAs from the original pools demonstrated similar phenotypic effects, strongly suggesting that attenuation of primary replication was due to knockdown of the target host factor rather than off-target effects. Asparagine synthetase (ASNS) was selected for further detailed characterization, as knockdown resulted in a substantial reduction in primary replication based on GFP expression, and has not previously been associated with HCMV or herpesviruses in general.

### ASNS is a crucial host factor for HCMV replication.

**FIG 3:**
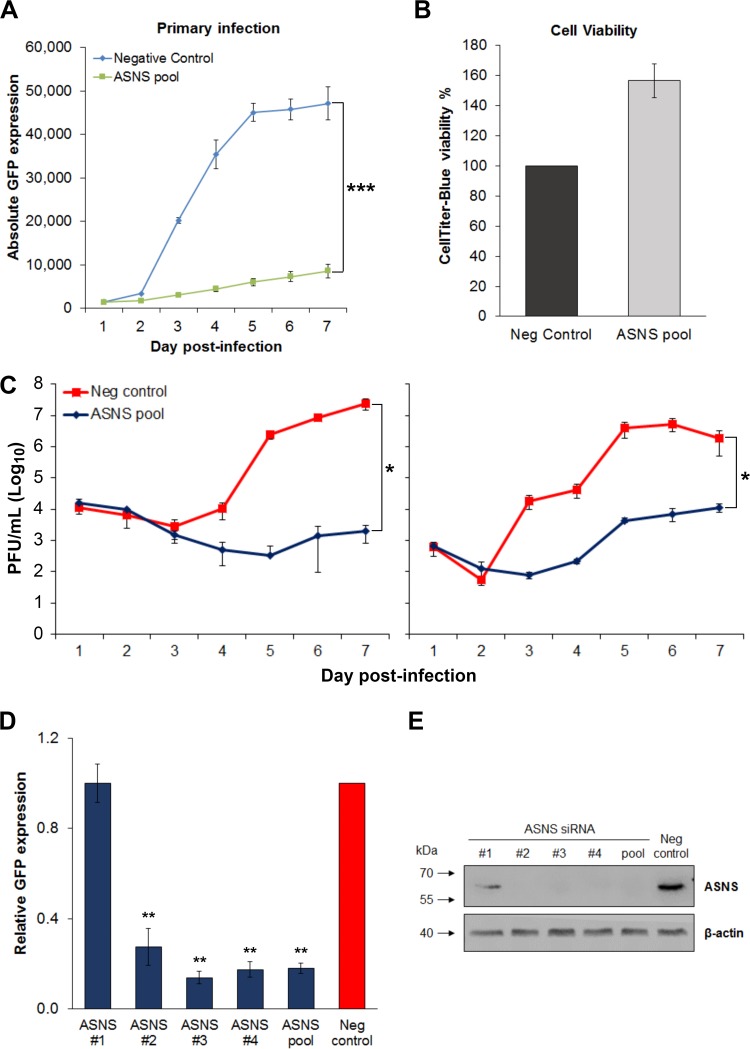
Asparagine synthetase (ASNS) is a novel host factor for HCMV replication. NHDF cells were transfected with an siRNA pool targeting ASNS or a scrambled negative control and infected 48 h posttransfection with HCMV TB40/E-GFP at an MOI of 5. (A) Knockdown of ASNS substantially decreased virus replication throughout the course of infection. Values are means ± standard deviations (error bars) (*n *=* *3). Two-way analysis of variance (ANOVA) was used to calculate statistical significance. ***, *P* < 0.0005. (B) CellTiter-Blue assay was performed at 7 days postinfection (DPI), and cell viability was calculated. *n *=* *3; error bars show standard deviations. (C) Cell-free (supernatant) and cell-associated virus (cells) were harvested at indicated time points, and virus levels were determined by plaque assay. *n *=* *2; error bars represent standard errors of the means. Two-way ANOVA was used to calculate statistical significance. *, *P* < 0.05. (D) NHDF cells were transfected with the four individual siRNAs targeting ASNS or a scrambled sequence (Neg control) and infected 48 h posttransfection with TB40/E-GFP at an MOI of 5. GFP levels were measured at 7 DPI to determine the effect of gene depletion on primary replication. *n *=* *3; error bars represent standard deviations. One-way ANOVA was used to calculate statistical significance between individual siRNA and the negative control. **, *P* < 0.005. (E) Western blot analysis showed ASNS protein levels following knockdown with individual siRNAs (siRNAs #1 to #4) and pool, along with negative control. Protein lysates were collected 4 DPI.

ASNS is the sole enzyme that catalyzes the biosynthesis of asparagine, by transferring the gamma amino group from glutamine to aspartate in an ATP-dependent manner. In the high-throughput screen, knockdown of ASNS inhibited HCMV primary replication, based on GFP expression, throughout the course of a single cycle infection (Fig. 3A). Inhibition was not due to siRNA cytotoxicity, as measured by CellTiter-Blue cytotoxicity assay (Fig. 3B), with cell viability increased after ASNS knockdown in infected cells compared to control transfected cells, possibly due to the inhibition of HCMV replication and subsequent decrease in cytopathic effect in infected cells.

To confirm that reduced GFP expression levels corresponded to reduced virus production, single-step growth curves were performed following knockdown of ASNS. Supernatant and cell-associated virus levels were determined following a high-multiplicity infection (MOI of 5) by plaque assay. In both pools, knockdown of ASNS resulted in a substantial decrease in virus production compared to control nontargeting siRNA-transfected cells, confirming the original observation based on GFP expression (Fig. 3C).

In order to confirm that the observed phenotype was due to specific protein knockdown and not due to off-target effects, we performed individual siRNA knockdown with four separate siRNAs from the pool. Three out of four siRNAs against ASNS showed the same phenotype as the reconstituted siRNA pool, whereas siRNA #1 showed no inhibition of primary replication (Fig. 3D). Western blot analysis with protein lysates collected at 96 h postinfection (HPI) revealed that siRNA #1 was less efficient at knocking down the protein, demonstrating a direct correlation between siRNA efficacy and attenuation of virus replication (Fig. 3E), confirming that the observed phenotype was due to specific knockdown of ASNS and not off-target effects.

### ASNS knockdown inhibits HCMV IE2 and subsequent gene expression.

**FIG 4:**
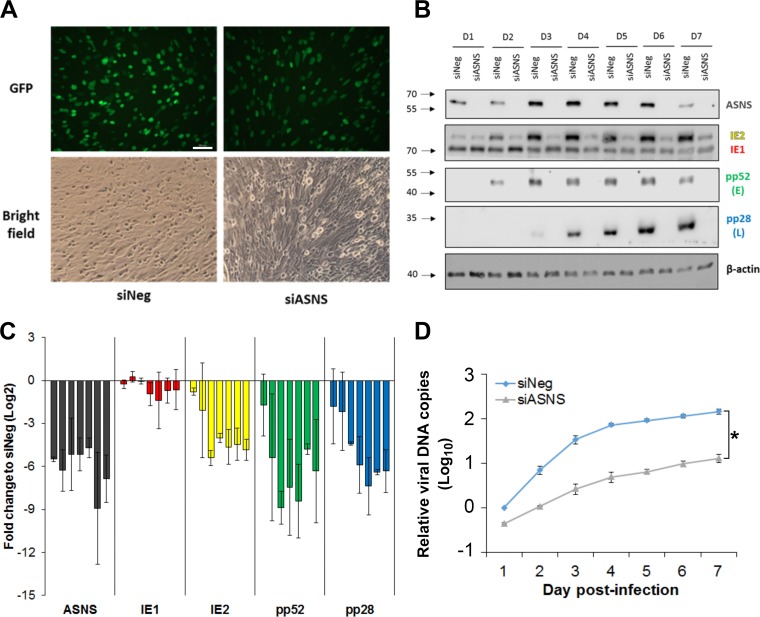
Knockdown of ASNS reduced IE2 expression and viral DNA amplification. NHDF cells were transfected with siRNA pool targeting ASNS (siASNS) or a scrambled negative-control siRNA (siNeg), followed by infection 48 h posttransfection with TB40/E-GFP at an MOI of 5. (A) Fluorescence images were taken at 1 DPI with ×100 magnification. Bar = 100 μm. (B) Total protein was harvested at the indicated time points (in days), and the levels of immediate early (IE1/2), early (pp52), and late (pp28) proteins were detected by Western blot analysis. (C) Quantification of Western blot data from panel B. The relative intensity of each band was normalized to β-actin compared to the siNeg value at day 1. Bars in each gene represent the differential expressions from 1 to 7 DPI (left to right). *n *=* *2. (D) Total genomic DNA was isolated at indicated time points, and viral genome levels were determined by qPCR. *n *=* *2; error bars show standard errors of means. Two-way ANOVA was used to calculate statistical significance. *, *P* < 0.05.

To determine where in the virus life cycle ASNS knockdown restricts virus replication, the associated phenotype was characterized in more detail. Knockdown of ASNS did not significantly affect viral entry or translocation of the genome to the nucleus, as the numbers of GFP-positive cells were equivalent between ASNS knockdown and control transfected cells at 24 h postinfection (Fig. 4A). Thereafter, we investigated the role of ASNS in HCMV gene expression in more detail. HCMV gene expression occurs in a temporal manner, with immediate early (IE), early (E), and late (L) gene expression phases. In order to identify the stage of the HCMV life cycle ASNS was involved in, we qualitatively measured the expression of IE (IE1/2), E (pp52), and L (pp28) genes by Western blot analysis following knockdown of ASNS (Fig. 4B and C). Following ASNS knockdown, IE1 expression was not drastically affected compared to the control nontargeting transfected cells. However, IE2 expression was substantially reduced, with subsequent E and L gene expression reduced to below the level of detection. While asparagine content of IE2 (4.31%) is higher than IE1 (2.85%), the difference is not substantial in the context of all HCMV proteins, with the levels ranging from 0 to 10.81% (see Fig. S1 in the supplemental material). The average asparagine content of HCMV proteins is 3.23%, slightly lower than the average of human proteins at 3.58%. Analysis of RNA levels by reverse transcription-quantitative PCR (RT-qPCR) shows that the loss of IE2 expression occurs at the transcriptional level, either through reduced IE promoter activity or a failure in splice switching between IE1 and IE2 (Fig. S2). Knockdown of ASNS is unlikely to have a direct effect on IE transcription and splicing, but rather the inhibition of virus replication occurs prior to the switch in differential splicing from IE1 to IE2, which likely reflects a major checkpoint for acute virus replication. Unsurprisingly, viral DNA levels were also significantly reduced following ASNS knockdown (Fig. 4D). These results indicate that knockdown of ASNS results in an early phenotype with inhibition of virus replication occurring after viral entry and translocation to the nucleus, but before IE2 gene expression and viral DNA amplification.

10.1128/mBio.01651-19.1 FIG S1 Asparagine content of HCMV proteins. Asparagine levels of HCMV proteins were calculated as a percentage of total amino acid content. Asparagine residues make up 2.85% of IE1 and 4.31% of IE2 protein (red dots labeled). The average Asparagine content of HCMV proteins is 3.23, whereas the average for human proteins is 3.58 (indicated by dotted lines in blot). Download FIG S1, TIF file, 2.5 MB. Copyright © 2019 Lee et al. 2019 Lee et al. This content is distributed under the terms of the Creative Commons Attribution 4.0 International license.

10.1128/mBio.01651-19.2 FIG S2 Knockdown of ASNS reduces accumulation of HCMV IE2 mRNA. NHDF cells were transfected with siRNA pool targeting ASNS (siASNS) or a scrambled negative control siRNA (siNeg), followed by infection 48 h posttransfection with TB40/E-GFP at an MOI of 5. Total RNA was extracted at the indicated time points, and the relative mRNA levels of IE1 and IE2 were quantified by RT-qPCR. GAPDH was used as a loading control. *n *=* *2; error bars showed standard deviations. Two-way ANOVA was used to calculate statistical significance. ***, *P* < 0.0005. Download FIG S2, TIF file, 2.1 MB. Copyright © 2019 Lee et al. 2019 Lee et al. This content is distributed under the terms of the Creative Commons Attribution 4.0 International license.

### Inhibition of HCMV replication is not due to a general loss of protein translation.

**FIG 5:**
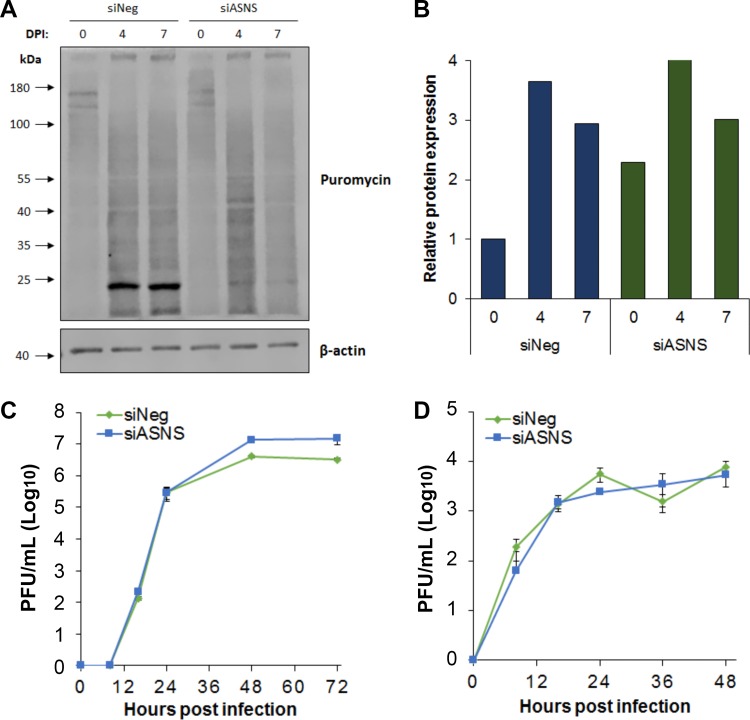
Inhibition of HCMV following ASNS knockdown is not due to loss of protein translation. (A) NHDF cells were transfected with siRNA pool targeting ASNS (siASNS) or a scrambled negative-control siRNA (siNeg) and then infected 48 h posttransfection with HCMV TB40/E-GFP at an MOI of 5. Cells were treated with puromycin at indicated times. Global protein translation levels were quantified based on puromycin incorporation by Western blot analysis (B) Quantification of Western blot data from panel A. The relative intensity of each band was normalized to β-actin compared to the siNeg value at day 0. (C and D) NHDF cells were transfected as described above and then infected with HSV-1 (C) or IAV (D) at an MOI of 0.1. Cells were harvested at the indicated times, and virus levels were quantified by plaque assay (*n* = 3; error bars show standard deviations).

As standard growth media do not contain asparagine, NHDF cells are dependent on ASNS for generation of *de novo* asparagine. Knockdown of ASNS would therefore be predicted to lead to asparagine depletion over time, ultimately impacting translation of proteins containing asparagine. However, the relatively early phenotype observed and the fact that IE1 protein expression levels were not dramatically affected while IE2 expression levels were substantially reduced suggest that the effect on HCMV replication following ASNS knockdown may not be due to a general loss of protein translation caused by asparagine deprivation. To directly measure protein translation levels in infected cells following ASNS knockdown, puromycin pulse studies were performed. Cells were transfected with siRNA against ASNS or a negative-control siRNA and infected with TB40E-GFP at an MOI of 5 at 2 days posttransfection. Puromycin was added at 0 (time of infection), 4, and 7 days postinfection (DPI) for 15 min, before total proteins were harvested. Global translation levels were measured by Western blot analysis using a puromycin-specific antibody. In addition to general labeling of proteins, a strong band was detected at approximately 24 kDa that is substantially reduced in the ASNS knockdown cells (Fig. 5A). Currently, the identity and relevance of this protein are not known; however, experiments are ongoing to identify the protein and determine whether it plays a direct role in ASNS-dependent HCMV replication. To avoid skewing the analysis, this band was omitted from the quantification analysis. Other than this band, the results show that relative protein translation levels were not reduced following ASNS knockdown in infected cells (Fig. 5A and B). This suggests that inhibition of HCMV replication from ASNS knockdown is not simply due to reduced protein translation due to the loss of available asparagine, but rather the virus is responding in a more indirect way to asparagine levels within the cell. Furthermore, knockdown of ASNS does not reduce replication of the related herpes simplex virus 1 (HSV-1) or influenza A virus (IAV). Following knockdown of ASNS, primary human fibroblast cells were infected with HSV-1 (strain C12) or IAV (strain A/PR/8/34 [PR8]) at 48 h posttransfection (Fig. 5C and D). In contrast to HCMV, HSV-1 and IAV replication were not significantly reduced, indicating that the cells are still capable of supporting robust virus replication and that the effect of ASNS knockdown is specific to HCMV.

### Knockdown of ASNS did not inhibit mTOR activity during HCMV replication.

**FIG 6:**
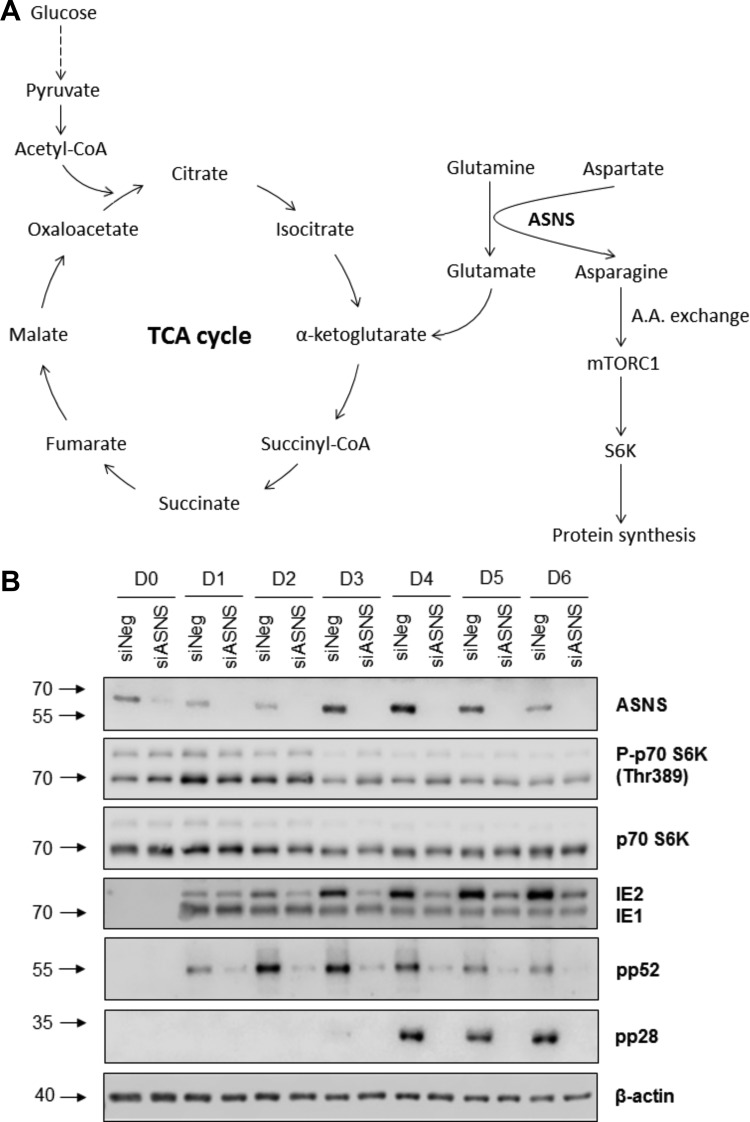
Asparagine depletion did not reduce mTOR signaling during HCMV replication. (A) A schematic diagram of host cell metabolism involving glycolysis, TCA cycle, and asparagine biosynthesis. Glucose is metabolized via glycolysis to produce acetyl coenzyme A (acetyl-CoA) which enters the tricarboxylic acid (TCA) cycle (also known as Krebs cycle). Glutamine is metabolized to glutamate, which can be further reduced to make α-ketoglutarate replenishing the TCA cycle. Generation of asparagine requires conversion of glutamine to glutamate. A.A., amino acid. (B) NHDF cells were transfected with siRNA against ASNS (siASNS) or a scrambled control sequence (siNeg), followed by infection 48 h posttransfection with HCMV TB40/E-GFP at an MOI of 5. Total protein was harvested at the indicated time points (in days). The levels of phosphorylated p70 S6K (P-p70 S6K), p70 S6K, immediate early (IE1/2), early (pp52), and late (pp28) proteins were determined by Western blot analysis. β-Actin was used as a loading control.

As a reduction in protein translation does not appear to explain the inhibition of HCMV replication following ASNS knockdown, asparagine levels may lead to an indirect inhibition of HCMV virus replication through signaling pathways that monitor amino acid levels. While previous studies have shown that HCMV can override cellular signals that would normally inhibit mTOR activation due to deprivation of essential amino acids, the effect of deprivation of nonessential amino acids has not been investigated. Asparagine has recently been shown to function as an amino acid exchange factor and an indirect regulator of mTOR signaling (Fig. 6A) (22). We therefore investigated mTOR signaling in ASNS-depleted cells during HCMV replication to determine whether depletion of the nonessential amino acid asparagine had an inhibitory effect on its activation. mTOR signaling was maintained upon ASNS knockdown, based on phosphorylation of ribosomal S6 kinase (S6K), which is the downstream effector of the mTOR complex (Fig. 6B). This suggests that the inhibitory effect of HCMV primary replication caused by ASNS depletion was not due to a loss of mTOR activation.

### Addition of asparagine does not fully rescue glutamine deprivation.

**FIG 7:**
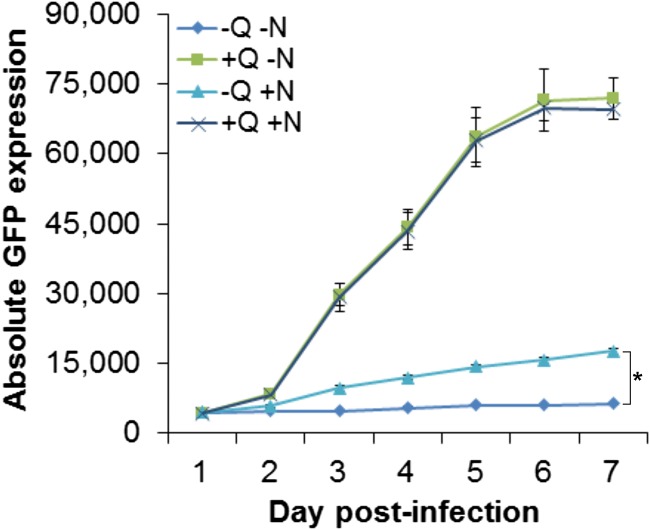
Asparagine supplementation partially rescued HCMV replication in glutamine-depleted cells. NHDF cells were grown in media with (+) or without (-) glutamine (Q) and 0.1 M asparagine (N) and infected with HCMV TB40/E-GFP at an MOI of 5 with indicated conditions. GFP fluorescence was monitored every 24 h for 7 days. *n *=* *3; error bars show standard deviations. Two-way ANOVA was used to calculate statistical significance and indicated as follows: *, *P* < 0.05.

Previous studies have shown that infection with HCMV results in increased glutamine metabolism and glutamine starvation results in attenuation of virus replication (7). During infection, increased glutamine metabolism compensates for the diversion of glucose from the TCA cycle. However, glutamine is also required for the *de novo* synthesis of asparagine by ASNS (Fig. 6A). A recent study demonstrated that the addition of asparagine could rescue vaccinia virus replication following glutamine deprivation (23). Therefore, we investigated whether loss of asparagine synthesis contributes to inhibition of HCMV replication following glutamine deprivation. NHDF cells were infected with HCMV in overlay media that contained asparagine (N) or glutamine (Q), both asparagine and glutamine, or neither. Dialyzed serum was used in all experiments to remove exogenous asparagine and glutamine. While the addition of asparagine resulted in a modest rescue of GFP signal, the results indicate that loss of asparagine synthesis is a minor contributing factor to the attenuation of virus replication through glutamine deprivation and that the loss of precursors for the TCA cycle likely represents the major contributing factor (Fig. 7).

### Asparagine depletion causes a reversible restriction of HCMV acute replication.

**FIG 8:**
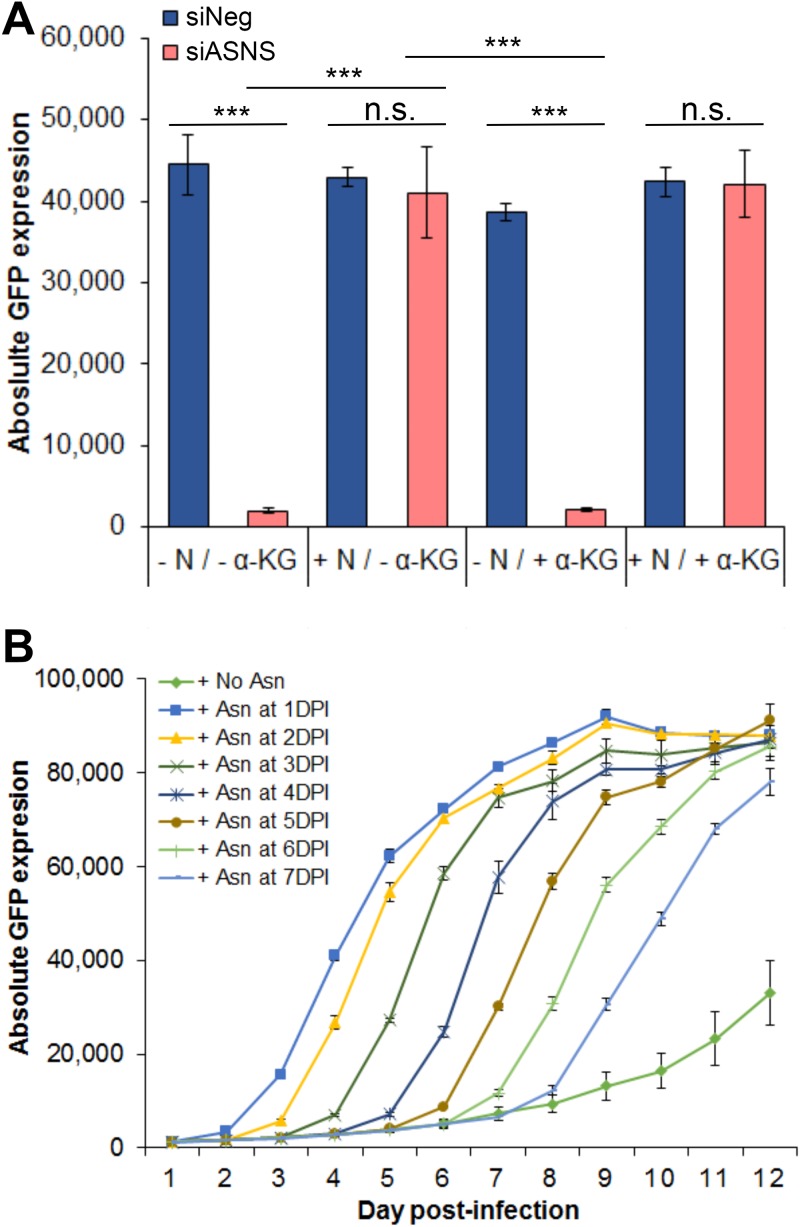
Asparagine deprivation established a reversible inhibition of HCMV infection. NHDF cells were transfected with siRNA pool targeting ASNS (siASNS) or a scrambled negative-control siRNA (siNeg), followed by infection 48 h posttransfection with TB40/E-GFP at an MOI of 5. (A) 0.1 mM asparagine or 7 mM α-ketoglutarate was supplemented in the medium at the time of infection with primary replication measured at 5 days postinfection based on GFP expression. (B) 0.1 mM asparagine was added at the indicated time points, and GFP fluorescence was monitored every 24 h for 12 days. The glutamine level remains unchanged at 0.876 g/liter. *n *=* *3; error bars showed standard deviations. n.s., not significant; ***, *P* < 0.0005.

Asparagine (Asn, or N) is a nonessential amino acid, meaning it can be produced enzymatically from precursors within cells. The primary fibroblast cells in this study are cultured in media that do not contain asparagine, leaving the cells dependent on this biosynthetic pathway. To determine whether the HCMV phenotype observed following ASNS knockdown was entirely dependent on asparagine deprivation, cells were incubated in media supplemented with 0.1 M asparagine 2 days prior to infection or at the time of infection and maintained throughout the time course. Again, dialyzed serum was used under all conditions. The results show that supplementation with asparagine completely rescued virus growth based on GFP reporter expression levels (Fig. 8A). This result indicates that the phenotype caused by knockdown of ASNS is due to a loss of available asparagine within the cell. Previous studies have shown that the addition of α-ketoglutarate, a primary substrate of the TCA cycle, could rescue virus replication following glutamine deprivation. However, virus replication was not rescued by supplementation with α-ketoglutarate following knockdown of ASNS, consistent with the virus phenotype being independent of the TCA cycle (Fig. 8A). Plaque assays were also performed and demonstrated that rescue of GFP signal corresponded to rescue of virus production (Fig. S3).

10.1128/mBio.01651-19.3 FIG S3 Supplementation of α-ketoglutarate does not rescue HCMV replication during asparagine deprivation. NHDF cells were transfected with siRNA pool targeting ASNS (siASNS) or a scrambled negative-control siRNA (siNeg), followed by infection 48 h posttransfection with TB40/E-GFP at an MOI of 5. 0.1 mM asparagine (N) or 7 mM α-ketoglutarate (α-KG) was supplemented in the medium at the time of infection. Supernatant was collected to determine relative PFU/ml by plaque assay. *n *=* *3; error bars showed standard deviations. One-way ANOVA was used to calculate statistical significance. n.s., not significant; *, *P* < 0.05; ***, *P* < 0.0005. Download FIG S3, TIF file, 1.3 MB. Copyright © 2019 Lee et al. 2019 Lee et al. This content is distributed under the terms of the Creative Commons Attribution 4.0 International license.

As supplementation of asparagine was shown to fully rescue primary replication when added at the time of infection, we wanted to determine whether primary replication could be rescued following extended knockdown of ASNS, when primary replication had stalled for multiple days. Remarkably, full primary replication could be initiated up to 7 DPI in ASNS knockdown cells with the addition of asparagine, indicating that while virus replication had stalled, infected cells remained viable, and the virus was maintained in a state whereby primary replication could be efficiently reinitiated (Fig. 8B). By 7 DPI, primary replication began to recover without the addition of asparagine to the media, likely due to the loss of siRNA-mediated ASNS knockdown. These results indicate that acute HCMV replication is highly dependent on cellular asparagine levels and that deprivation results in inhibition of the replication cycle prior to DNA amplification and IE2 expression. However, the virus maintains the ability to resume virus replication when asparagine is supplied and reaches peak levels of replication based on GFP reporter gene expression, even following prolonged conditions of asparagine deprivation.

## DISCUSSION

Identification and characterization of novel host-virus interactions provide valuable insights into how viruses replicate and can furnish information about the functions of basic cell biology and potentially identify targets for antiviral interventions. Using a high-throughput siRNA screen targeting 6,881 genes, we identified multiple host factors important for HCMV replication, including ASNS, and demonstrate that the nonessential amino acid asparagine is required for HCMV replication at an early stage. Despite ASNS knockdown, global protein translation levels were maintained. Furthermore, given that the block in virus replication occurs relatively early in infection and knockdown cells could still fully support replication of HSV-1 and IAV, the loss of replication may not be simply due to loss of protein production, but rather indicates that asparagine levels feed into a signaling pathway that influences replication of HCMV. While asparagine depletion had little effect on HSV-1 or IAV replication, a recent report demonstrated that, like HCMV, vaccinia virus (VACV) is also highly dependent on asparagine levels and knockdown of ASNS resulted in attenuation of virus replication (23). Interestingly, despite the similar phenotype, there appear to be differences in the effects of asparagine depletion on the two viruses. For example, addition of asparagine can fully rescue attenuation of VACV following glutamine deprivation, indicating that increased glutamine metabolism in VACV-infected cells is necessary for asparagine synthesis, rather than contributing precursors for the TCA cycle. Furthermore, attenuation appears to be due to a loss of protein production in VACV-infected cells. Therefore, while asparagine deprivation has similar effects on both viruses, the mechanisms underlying asparagine requirement may be different for the viruses.

There is an increasing appreciation that amino acids are not just building blocks for protein production, but awareness that amino acids participate in many signaling pathways within a cell, affecting protein translation, cell cycle, and even apoptosis (24–27). While mTOR is part of a major signaling pathway affected by amino acid levels, our data indicate that knockdown of ASNS in infected cells does not block HCMV activation of mTOR; therefore, loss of mTOR signaling does not account for attenuation of virus replication.

Inactive ASNS has been shown to affect cell cycle (27). Infection with HCMV causes cell cycle arrest between G_1_ and S phase, and studies have shown that virus replication is blocked during other phases of cell cycle due to a failure in immediate early gene expression (28, 29). However, as loss of asparagine synthesis results in a block in the cell cycle at the G_1_ phase, it is unlikely that alteration of cell cycle by ASNS knockdown explains the attenuation of virus replication. Further studies will be required to determine the precise mechanism by which asparagine deprivation results in HCMV attenuation and potential signaling pathways that may be involved.

HCMV infection remains an important clinical issue, both in immunocompromised individuals and during pregnancy, where spread of the virus to the fetus can lead to serious developmental pathologies. There is currently no effective vaccine, and available antiviral therapies have significant issues, including side effects and development of resistance. Therefore, the generation of new treatments against HCMV is required.

Very few antivirals have been developed for use against HCMV since the licensing of ganciclovir, and of these, the same viral genes are often targeted, reducing the effectiveness of these drugs against resistant strains. An alternative strategy for the development of novel antivirals involves targeting of host genes or metabolites required by the virus for successful replication. Development of resistance against drugs that target host genes would be far more complex, as the virus would have to gain mutations that would compensate for the loss of a required cellular factor. In many cases, such mutations may not exist.

We have shown that reducing available asparagine levels has a profound inhibitory effect on HCMV replication, suggesting that this may be a potential strategy for limiting HCMV replication in patients. A recent study demonstrated that reducing asparagine levels in mice through treatment with l-asparaginase or through dietary restriction reduced metastatic spread of tumor cells (30). As discussed, tumor cells demonstrate metabolic alterations similar to HCMV-infected cells, with increased anaplerosis and dependence on asparagine (7). While cells are able to make *de novo* asparagine through ASNS, this study suggests that under certain physiological conditions, cells still require free exogenous asparagine, especially cells with a high asparagine dependence. Therefore, reducing available asparagine levels through treatment with l-asparaginase or by dietary restriction may be an effective clinical approach for treating HCMV infection in high-risk patients. Temporary dietary restriction would be a particularly attractive approach given the limited likelihood of serious side effects. In addition to reducing exogenous asparagine level by l-asparaginase, l-albizziine is a competitive inhibitor against ASNS, albeit the functional concentration is in the millimolar range, and it exhibits reactivity against other enzymes such as glucosamine synthetase and glutaminase (23, 31, 32). In addition, amino sulfoximine derivatives have been shown to inhibit ASNS, although issues such as poor cell permeability reduce the viability of these approaches (33–35). While the approach of reducing intracellular and extracellular asparagine levels would not eliminate the virus, as demonstrated by rescue of virus replication days later with exogenous asparagine, subduing virus replication in combination with other antiviral drugs may still be therapeutically beneficial.

Finally, it will be interesting to determine the potential impact of asparagine levels, and amino acid metabolism in general, on the establishment, maintenance, and reactivation of HCMV during latency. Previous studies have demonstrated the importance of glutamine and asparagine metabolism during herpesvirus latency. Similar to HCMV-infected cells, endothelial cells latently infected with Kaposi’s sarcoma herpesvirus (KSHV) are dependent on glutaminolysis, with glutamine deprivation resulting in increased cell death and apoptosis (3). However, glutamine deprivation can be fully rescued by the addition of asparagine but not α-ketoglutarate, with asparagine being important for purine and pyrimidine synthesis (36). In contrast to these studies, dietary supplementation of glutamine reduced HSV reactivation in mouse and guinea pig latency models. This effect was linked to increased gamma interferon (IFN-γ)-producing CD8 T cells, suggesting that the effects of amino acid metabolism on herpesvirus replication may be more complex *in vivo* due to effects on the host immune response (37).

To our knowledge, the roles of amino acid metabolism and in particular asparagine metabolism have not been investigated in latent models of HCMV infection. A recent paper reported effects on amino acid metabolism during acute HCMV replication in primary fibroblast cells (8), and it is clear that the virus modulates amino acid levels and metabolism, including induction of ASNS as shown here. However, it will be of interest to determine asparagine levels and levels of amino acids in general during models of HCMV latency and reactivation to establish whether there is any link between regulation of latency and amino acid metabolism.

## MATERIALS AND METHODS

### Cell culture and virus infection.

Normal human dermal fibroblast (NHDF) (Gibco) cells were maintained in Dulbecco’s modified high-glucose medium (DMEM) (Sigma) supplemented with 10% fetal bovine serum (FBS) (Gibco) and 1× penicillin-streptomycin-glutamine (Gibco). A low-passage-number HCMV strain TB40/E-GFP, which is engineered to constitutively express GFP from an SV40 promoter at the intragenic region between TRS1 and US34, was obtained from F. Goodrum (13) and used for all experiments.

### siRNA screening.

The Dharmacon SMARTpool human druggable genome (G-004600-05), cell cycle (G-003250-02), and protein kinase (G-003500-02) siRNA libraries against 6,881 gene targets were prepared in the 96-well format at a concentration of 3 μM (diluted in Thermo Scientific siRNA buffer) at the Division of Infection and Pathway Medicine, University of Edinburgh, followed by the set-up of 384-well master plates at a concentration of 311 nM, using a RapidPlate 384 liquid handling robot (Qiagen). The complete protocol can be found in reference 38. Low-passage NHDF cell suspension (at 3,000 cells per well) were reverse transfected with siRNA and Lipofectamine RNAiMAX (Invitrogen) using MultiDrop 384. At 48 h posttransfection, media were removed, and cells were infected with strain TB40/E-GFP at a multiplicity of infection (MOI) of 5. Absolute GFP expression is measured by Cytation 3 cell imaging multimode microplate reader (BioTek) every 24 h for 7 days, and relative GFP expression (or replication ratio) was calculated by dividing the absolute GFP expression of infected cells with the corresponding gene knockdown by the nontargeting siRNA control sample.

### Cell viability assay.

Two cell viability assays were performed at 7 days postinfection (DPI). Media in plates were removed, and the CellTiter-Blue reagent and fresh media were added by MultiDrop 384. After incubation for 1 to 4 h, the fluorescence at 560/590 nm was measured by a Cytation 3 cell microplate reader (BioTek). The relative cell viability was normalized to the reading of control nontargeting siRNA-transfected cells.

### Amino acid depletion and supplementation.

The DMEM (catalog no. D5796; Gibco) used for normal cell culture contains a total of 0.876 g/liter of l-glutamine and no l-asparagine. An alternative DMEM without l-glutamine (catalog no. D5030; Gibco) was used to starve cells without l-glutamine and l-asparagine. In preparing DMEM without l-glutamine, equal amounts of sodium bicarbonate (3.7 g/liter) and glucose (4.5 g/liter) to DMEM (D5030) were added. For l-asparagine supplementation, 0.1 mM l-asparagine was added, either in DMEM D5030 (to make –Q+N) or in DMEM D5796 (to make +Q+N). For α-ketoglutarate (α-KG) supplementation, 7 mM α-ketoglutarate at pH 7.2 was added to DMEM D5796. All media were supplemented with dialyzed FBS (Labtech).

### Western blot analysis.

Following transfection, cells were harvested at 0 to 7 DPI in radioimmunoprecipitation assay (RIPA) buffer containing protease and phosphatase inhibitor cocktails (Roche). Protein concentrations were determined by bicinchoninic acid (BCA) assay (Thermo Fisher) following the manufacturer**’**s protocol. The proteins were separated on 10% SDS-PAGE gels and transferred to nitrocellulose membranes by wet transfer (20% methanol). The membranes were blocked with 5% milk in Tris-buffered saline (TBS) and probed with antibodies to HCMV IE1 and IE2 (MAB-8131; Merck Millipore; diluted 1/5,000), pp52 (sc-56971; Santa Cruz Biotechnology; 1/1,000), pp28 (sc-69749; Santa Cruz Biotechnology; 1/1,000), ASNS (catalog no. 14681-1-AP; Proteintech; 1/1,000), p70 S6 kinase (S6K) (Cell Signaling Technology; 2708; 1/1,000), phospho-Thr389 p70 S6K (catalog no. 9234; Cell Signaling Technology; 1/500), and β-actin (ab8227; Abcam; 1/2,500). Secondary antibodies conjugated to horseradish peroxidase (HRP) (Thermo Fisher) or IR800 and IR680 dye (Li-Cor) were used, and blots were imaged by Li-Cor Odyssey Fc imaging system. Quantification was performed with Li-Cor Image Studio Lite software.

### qPCR.

DNA was purified using a DNeasy blood and tissue kit (Qiagen) and quantified with a NanoDrop spectrophotometer. The SensiFAST SYBR Hi-ROX kit (catalog no. BIO-92020; Bioline United Kingdom) and custom gene-specific primer sets were used to assay 20 ng DNA per reaction for HCMV gB (UL55) and glyceraldehyde-3-phosphate dehydrogenase (GAPDH) using the following primers: GAPDH DNA, 5′-GATGACATCAAGAAGGTGGTGA and 5′-CCTGCACTTTTTAAGAGCCAGT; HCMV gB (UL55), 5′-TAGCTACGACGAAACGTCAAAA and 5′-GGTACGGATCTTATTCGCTTTG. Results were normalized to GAPDH DNA levels and then to scrambled negative-control siRNA (siNeg) levels at 1 day postinfection (DPI) by the ΔΔ*C_T_* method (*n *=* *2; error bars represent standard errors of the means).

### RT-qPCR.

Total RNA was extracted using TRIzol reagent following the manufacturer’s protocol. RNA was treated with DNase (Turbo DNA-free kit; Ambion) and reverse transcribed with poly(T) and random primers using high-capacity cDNA reverse transcription kit (Invitrogen). Real-time qPCR was performed on Rotor gene 3000 (Corbet Research) using TaqMan assays with custom primer/probes for HCMV IE1 and IE2 as previously described (14).

### STRING analysis.

Functional annotation clustering was performed in the free software, Cytospace, with stringApp. The top 115 proviral and antiviral hits were analyzed, and only interactions with a confidence score of 0.8 or above were shown. To create the network view, each gene was assigned to the cluster with the highest enrichment where the gene was present in the highest fraction of individual clusters. For visualization purposes, the interactions of a gene with multiple genes in the same cluster (such as the interactions of TAF1 with POLR2A, POLR2B, and POLR2G) were removed. Genes that showed no interaction on the network were depicted by diamonds (Fig. 2). The effect of gene knockdown on relative GFP expression of virus replication was indicated in red (reduction) or green (enhancement) in the top right corner of the gene. The functional annotation clusters were arranged in their approximate cellular locations in the final image. Some smaller annotation clusters and unconnected genes (43 genes) were left out due to space limitation.

### Bioinformatics and statistical analysis.

siRNA screen data were analyzed using Microsoft Excel and its data analytic tools. R studio was used to analyze the correlation between triplicate screens, using the *psych* package. Asparagine percentages of HCMV proteins were calculated based on the amino acid sequences downloaded from UniProt.org (UniProt accession no. UP000008991). Two-tailed homoscedastic Student’s *t* test was applied to calculate the *P* values of the effect of individual gene depletion on HCMV replication.
