# Supplementary figures and images for: Asparagine Deprivation Causes a Reversible Inhibition of Human Cytomegalovirus Acute Virus Replication
Source: mBio. 2019 Oct 8;10(5):e01651-19. doi: 10.1128/mBio.01651-19 (PMC6786868; doi:10.1128/mBio.01651-19)

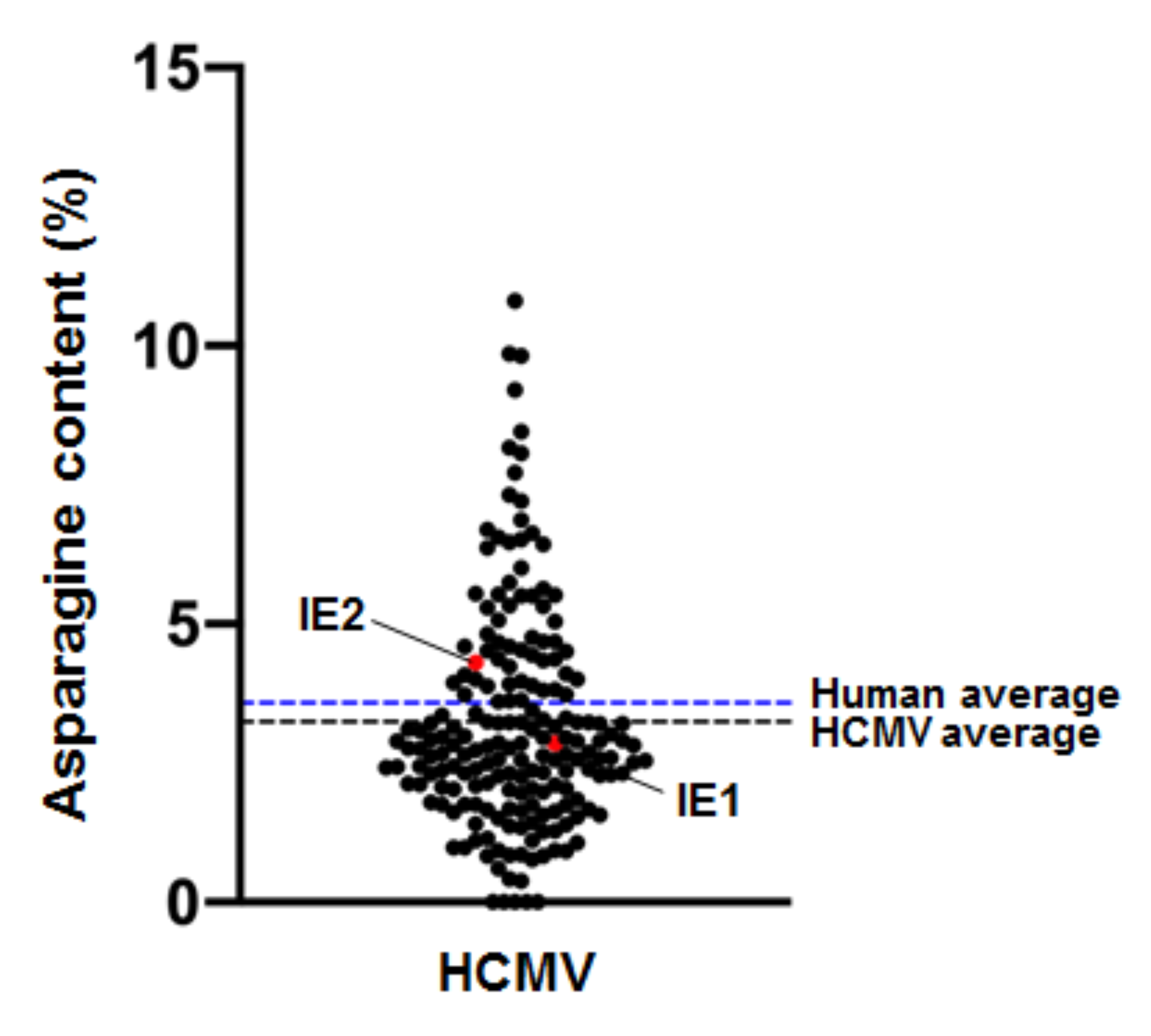

Supplement: FIG S1 [file mBio.01651-19-sf001.tif]

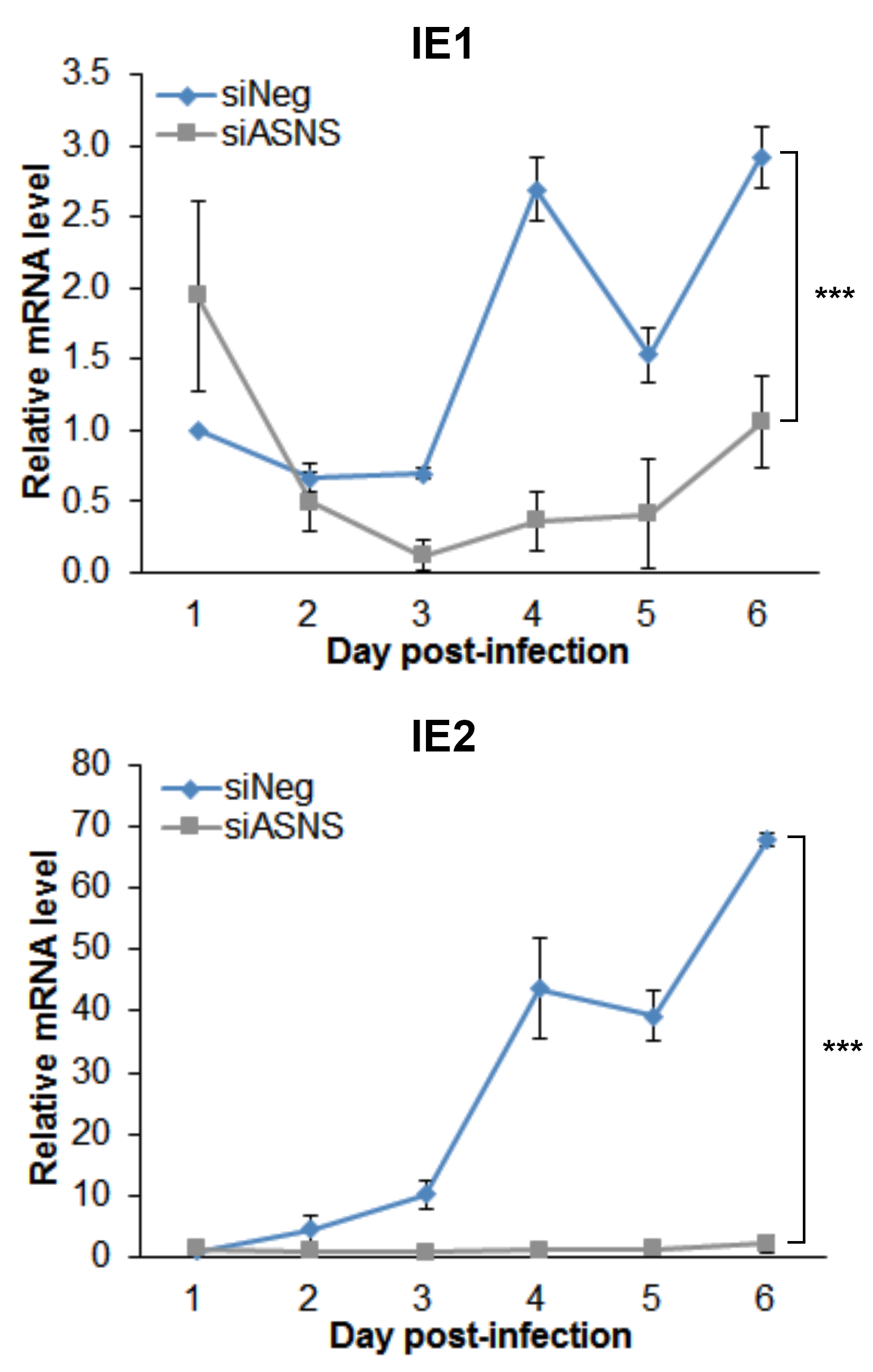

Supplement: FIG S2 [file mBio.01651-19-sf002.tif]

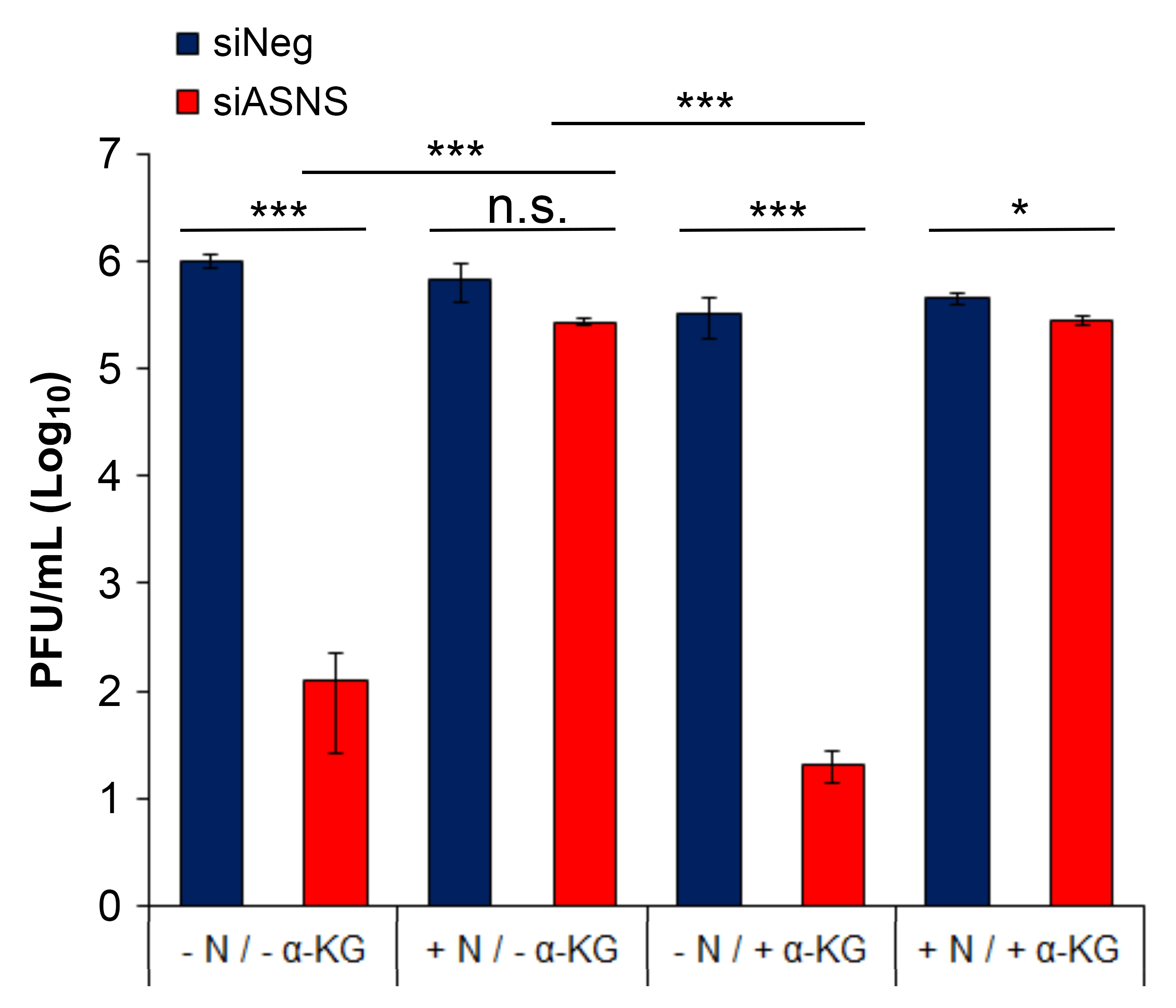

Supplement: FIG S3 [file mBio.01651-19-sf003.tif]
